# Let's Make Gender Diversity in Data Science a Priority Right from the Start

**DOI:** 10.1371/journal.pbio.1002206

**Published:** 2015-07-27

**Authors:** Francine D. Berman, Philip E. Bourne

**Affiliations:** 1 Department of Computer Science, Rensselaer Polytechnic Institute, Troy, New York, United States of America; 2 Office of the Director, National Institutes of Health, Bethesda, Maryland, United States of America

## Abstract

The emergent field of data science is a critical driver for innovation in all sectors, a focus of tremendous workforce development, and an area of increasing importance within science, technology, engineering, and math (STEM). In all of its aspects, data science has the potential to narrow the gender gap and set a new bar for inclusion. To evolve data science in a way that promotes gender diversity, we must address two challenges: (1) how to increase the number of women acquiring skills and working in data science and (2) how to evolve organizations and professional cultures to better retain and advance women in data science. Everyone can contribute.

## Abstract 2

Data science is accelerating innovation, and the best and the brightest from both genders are needed to achieve its potential. This Perspective discusses what you can do to advance the field.

Every March we celebrate both International Women’s Day and Women’s History Month. These annual celebrations remind us that through our current individual and collective behavior, all stakeholders can influence how gender-diverse our future history is likely to be. This is especially important in data science, an emerging science, technology, engineering, and math (STEM) field that is a critical driver for 21st century innovation.

Data science focuses on the extraction of knowledge from data. It is a STEM discipline, but requires skills not yet widely taught in STEM disciplines: Skills in managing large datasets, novel analysis and inference approaches, rigorous statistical analysis, new ways to convey outcomes, and more. A recent McKinsey Report [1] indicates that the United States alone will need 1.5 million more data-savvy professionals and 140,000–190,000 more professionals with deep analytic skills by 2020. Helping to create and nurture a broad pool of individuals with data science skills is critical to addressing this growing need and will require intentional action.

The emergent field of data science offers the opportunity to narrow the gender gap in STEM (in which only 13% of the engineering workforce and 25% of the computer and mathematical sciences workforce are women [2]) by making diversity a priority early on. In addition to this being the right thing to do, it is the smart thing to do: studies show that companies with employees characterized by diverse inherent traits (traits you were born with) and acquired traits (traits you gain from experience) are 45% more likely to report a growth in market share over the previous year, and 70% more likely to report capture of a new market [3]. Companies with diverse executive boards show higher returns on equity [4]. In short, diversity is a competitive asset in the private sector. In addition, increased diversity in STEM fields, including data science, is a national research and education priority [5].

What better time, with increased focus on data science in the public sector, emerging educational curricula and focus within universities, and greater need within the private sector, to foster greater inclusivity and gender diversity? What can we do now to grow data science in a way that reflects the gender diversity and potential for innovation of the greater society?

To evolve data science in a way that makes it a rewarding and sustainable career choice for women, we need to address two challenges: how can we increase the number of women acquiring skills and working in data science, and how can we evolve organizations and professional cultures to better retain and advance women in data science?

## How Can We Increase the Number of Women in Data Science?

We can’t have a pipeline of women in data science careers without attracting them to the field in the first place. Inclusion of data science within educational curricula, STEM programs, and outreach can leverage existing efforts and introduce key skills into the mix that can help build the data science workforce. This is especially important in primary and secondary school, when students are evolving their STEM skills and developing professional interests. Moreover, we need to get the word out that data scientists work in many fields—from determining the best content to deliver for Netflix, to helping the Centers for Disease Control and Prevention track flu epidemics, to helping retailers determine how to market to customers. Data science careers abound in education, government, for-profit companies, and non-profit organizations, and data science is increasingly applied in virtually every domain. Its interdisciplinarity and broad application makes it attractive to many different people with diverse interests, and it needs diversity in its practitioners.

To broaden the pipeline, we also need to create positive social messages around women in data science. In STEM, the social messages that make it more acceptable than it should be for girls and women to be “bad at math” have served to limit women’s self-perception, training, and options. Studies show that girls internalize pervasive societal stereotypes about math ability from an early age, prior to differences seen in actual math performance and ability [6] and these stereotypes affect math performance [7], mathematics course taking, and career pursuits [8].

How can we create social messages that indicate that it’s cool for women to be “good at data science”? What if we could get the social messages right for data science from the start? How would that change the way we think about professional options, potential candidate pools, or the gender balance for data science classes or jobs? How would that advance the field?

To do this requires not just education and skill-building but cultural change. Positive social messages and role models can make a difference in dispelling negative stereotypes [9], as can action and support on the part of organizations and stakeholders—schools and academic institutions, teachers and faculty, funders, and popular media. For the messages to be credible, however, women and girls will need evidence that they can be successful in careers as data scientists.

## How Do We Ensure That Data Science Careers Are Fulfilling for Women?

If we are successful in getting women into data science, how do we retain them and provide adequate opportunities for advancement? This is a current challenge in the field of information technology, in which there is much discussion of “brogramming culture” [10], a professional “chilly climate,” and “glass ceiling” issues [11], despite attempts by both women and men to make professional cultures more diverse and inclusive. Data demonstrate that 56% of women in technology have left their employers by the time they reach the mid-level of their careers. Of those women, only half remain in technical positions [12].

At last year’s Grace Hopper Celebration of Women (http://gracehopper.org/), an internationally recognized conference for women in technology, more than 8,000 women in technology heard keynote speeches by the US Chief Technology Officer Megan Smith, Turing Award Winner Shafi Goldwasser, and others. The focus of the working sessions was recruitment, advancement, and retention of women in information technology writ large, including data science. It was clear from the broad scope of experience at all levels that skill building is critical for women (and men) in the workforce, but culture change is the other part of the equation. A key lesson from the conference was that for the data science culture to be open and welcoming to women, we must proactively develop the needed professional structures, curricula, training, advancement mechanisms, and support that strategically and intentionally promote gender diversity.

## What Can Each of Us Do to Bring About Culture Change?

At the end of the day, culture change is created by the individual actions of many. To support the prioritization of gender diversity in data science, changes in organizational culture are needed to complement individual action. Box 1 provides some simple actions that each of us can take. We encourage readers to add their own ten actions to this list, adopt those actions, and help accelerate cultural transformation through individual behavior.

**Box 1. Ten Simple Rules for Increasing Gender Diversity in Data Science**

**Get data science culture right at our places of employment:**

1. Foster a recruitment process that seeks out diverse candidate pools and engages in targeted and intentional outreach efforts that attract a diverse applicant pool.
2. Monitor and promote pay equity.
3. Develop organizational mechanisms for promoting diversity in which success of such efforts can be measured and rewarded.
4. Provide leadership opportunities for women, promote their efforts, and help women identify advancement opportunities.
5. Make diversity a strategic priority and expect those who work for and/or with us to do so as well.

**Become an activist for women’s representation in data science:**

1. Put women colleagues and students up for awards and recognitions. Share their work with colleagues. Provide mentorship that helps them navigate the pathways to success. Help clear those paths.
2. Raise awareness of diversity. If you are asked to present, be on a panel, or serve on a committee, ask if there are (other) women participating. If not, suggest names of women to invite. (Bonus points for not accepting the response that finding good women candidates is hard.) This approach is particularly effective when both men and women ask these questions.
3. Help create positive language and social expectations around data science: assume that women will be part of the process and part of the leadership. Spread the word that women’s leadership belongs in data science. Position and highlight the expertise of women in the field and include their voices.
4. Send colleagues and students to the Grace Hopper Celebration of Women (and go yourself). Encourage them to utilize this meeting to expand their networks, advance their professional success, and develop mentors, colleagues and friends in data science.
5. Focus on daily actions and interactions. Make sure that women’s contributions are valued and heard. Model inclusive behavior and hold yourself and others accountable.

When we change the language, reward mechanisms, and assumptions, we can improve the culture. When we improve the culture, we can evolve data science as an open, gender-diverse field that can better drive current and future innovation. So let’s capitalize on the tremendous opportunity that data science has to reduce the gender gap in STEM. Let’s create a history that will lead to greater success and one that we will be proud to have been part of.

**About the Authors**

**Francine Berman** is Chair of the Research Data Alliance/United States, Hamilton Distinguished Professor of Computer Science at Rensselaer Polytechnic Institute, and Chair of the Board of Trustees of the Anita Borg Institute for Women and Technology.

**Philip E. Bourne** is Associate Director for Data Science, the National Institutes of Health, and Founding Editor-in-Chief of *PLOS Computational Biology*.
